# Assessment of Different Levels of Blackcurrant Juice and Furcellaran on the Quality of Fermented Whey-Based Beverages Using Rheological and Mechanical Vibration Damping Techniques

**DOI:** 10.3390/foods13121855

**Published:** 2024-06-13

**Authors:** Anita Rejdlová, Martin Vašina, Eva Lorencová, Lumír Hružík, Richardos Nikolaos Salek

**Affiliations:** 1 Department of Food Technology, Faculty of Technology, Tomas Bata University in Zlin, Nám. T. G. Masaryka 5555, 760 01 Zlin, Czech Republic; a_rejdlova@utb.cz (A.R.); lorencova@utb.cz (E.L.); rsalek@utb.cz (R.N.S.); 2 Department of Physics and Materials Engineering, Faculty of Technology, Tomas Bata University in Zlin, Vavrečkova 5669, 760 01 Zlin, Czech Republic; 3 Department of Hydromechanics and Hydraulic Equipment, Faculty of Mechanical Engineering, VŠB-Technical University of Ostrava, 17. listopadu 2172/15, 708 00 Ostrava-Poruba, Czech Republic; lumir.hruzik@vsb.cz

**Keywords:** fermented whey-based beverages, water kefir starter culture, furcellaran, rheological properties, mechanical vibration damping

## Abstract

In the current study, fermented whey-based beverage models with different levels of blackcurrant juice (0; 10; 20; 100% (*w*/*w*)) and furcellaran (0.25% and 0.50% (*w*/*w*)) were produced and evaluated. Physicochemical, rheological, mechanical vibration damping, and sensory analyses were performed. During fermentation (48 h), the values of pH, density, and total soluble solids decreased. On the other hand, the ethanol content during fermentation increased up to a final content in the range of 0.92–4.86% (*v*/*v*). The addition of furcellaran was effective in terms of sediment content decrease to a level of 0.25% (*w*/*w*). In general, the samples exhibited non-Newtonian pseudoplastic behaviour. The sensory analysis revealed that the sample with a composition of 20% (*w*/*w*) blackcurrant juice and 0.50% (*w*/*w*) furcellaran received the highest score.

## 1. Introduction

The popularity of functional foods among consumers is steadily increasing, mainly due to their proven positive effects on the human body [1]. These functional foods may include fermented whey-based beverages [2]. Whey is produced as a by-product of the dairy industry and can be used in the food industry or as an ingredient of animal feed. However, the high volume of whey production and high organic content may lead to environmental issues. The inappropriate disposal of whey (by spreading on the soil) can negatively affect crop yields or cause problems in wastewater treatment plants (disposal in sewers) [2,3]. Whey has a high nutritional value; it contains BCAA (branched-chain amino acid, leucine, isoleucine, and valine), which promote, for example, muscle fibre regeneration. Moreover, whey is also a source of lactose, vitamins, and minerals [4].

In general, fermentation increases the nutritional value of products and also the bioavailability of nutrients [5]. In particular, it is the content of the above-mentioned substances that makes whey a suitable substrate for the production of value-added foods such as beverages, protein products, hydrolysates, infant formula, or pharmaceuticals and supplements. Up to 50% of the world’s whey production is processed in this way [6,7]. There are many types of whey drinks, for example fermented, alcoholic, carbonated (Rivella-type) or flavoured [8]. Additionally, lactic acid bacteria (LAB) starter cultures can be used in fermentation where kefir starter cultures are also suitable. At the same time, water kefir starter cultures, which are able to ferment whey, can also be utilised in whey fermentation [9,10,11].

Kefir starter cultures contain both LAB and yeasts, as well as acetic acid bacteria [12]. However, the representation of each microorganism causes different fermentation conditions. In particular, water kefir grains contain dextran, which is a glucose polymer, mainly composed of linear α-D-1,6-linked with a low percentage of α-1,3-linked side chains. The appearance of the grains is gelatinous and transparent, they may be yellowish to brown in colour; however, the colour is influenced by the substrate colour (e.g., the colour of the fruit/vegetable used). Moreover, water kefir grains are irregular and range in size from millimetres to a few centimetres [13,14].

Different forms of whey are used in the production of fermented whey drinks. Due to the high water, carbohydrate and protein contents of fresh whey, there is a risk of contamination with undesirable microbiota. For this reason, heat treatment is used before fermentation. In the case of powdered whey, the high mineral content may cause an undesirable salty and/or sour flavour. Furthermore, various raw materials may be added to the whey to improve the sensory properties. These include, for example, various milk components, cereals and possibly fruit/vegetable juices. At the same time, the addition of the latter ingredients also increases the nutritional value of the final whey-based beverage [5,6,15]. The blackcurrant (*Ribes nigrum*) fruit is characterised by its high content of biologically active substances, being an important source of dietary fibre, vitamin C and polyphenols (anthocyanins, phenolic compounds, flavanols, flavonols and proanthocyanidins). However, the polyphenol content may contribute to bitterness, astringency, colour and oxidative stability [16,17]. Blackcurrants have been found to help lower blood sugar (glycaemia) and are therefore, suitable for people with type *2* diabetes mellitus. Consumption of blackcurrants also reduces the risk of cardiovascular disease and cancer due to their phytochemical content, but direct consumption is rare, so blackcurrants are processed industrially into juices, syrups and other products [18,19].

The disadvantage of combining whey and fruit/vegetable juice is possible sedimentation, due to the high dry matter content and protein interactions with the fruit components. This would lead, among other things, to the aggregation of whey proteins, which subsequently sediment. Thus, it is the high sediment content that causes these beverages to be less acceptable by consumers [20,21]. Therefore, additives, including hydrocolloids, are used to eliminate this problem. Hydrocolloids (biopolymers; mainly polysaccharides and proteins) are widely used in the food industry due to their functional properties (thickening, gelling, emulsifying, stabilizing). The addition of hydrocolloids also influences the rheological properties (viscosity), leading to an improvement in the organoleptic properties of the product [22,23]. Furcellaran (also known as “Danish agar”) is obtained from the red algae *Furcellaria lumbricali* and is a sulphated polysaccharide composed of galactose and 3,6-anhydrogalactose esters. Furcelleran is used in the dairy industry in the production of puddings, milkshakes, ice cream mixtures [24,25].

A very limited number of publications focusing on the production of fermented whey-based beverages enriched with fruit juices is available in the scientific literature. Up to now, the utilisation of a water kefir starter culture for the development of fermented whey-based beverages has not been thoroughly examined. In addition, there is currently a lack of research evaluating the functional properties of fermented whey-based beverages through the application of rheological and mechanical vibration damping techniques. In the current study, sample models with different proportions of whey and blackcurrant juice (100:0, 90:10, 80:20, and 0:100) were manufactured. Two concentrations of furcellaran (0.25% and 0.50% *w*/*w*) were applied to the samples. The objective of the current work was to investigate the influence of blackcurrant fruit juice and furcellaran addition on the physicochemical, rheological, mechanical vibration damping, and sensory properties of the whey-based beverage models during fermentation (48 h) at a temperature of 25 ± 1 °C.

## 2. Materials and Methods

### 2.1. Materials

Fermented whey-based beverage models were prepared using whey powder (Mogador, s.r.o, Otrokovice, Czech Republic) with a chemical composition of: 0.41% (*w*/*w*) fat, 76.0% (*w*/*w*) carbohydrates, and 13.0% (*w*/*w*) protein (information obtained from the packaging). Furthermore, the beverage models contained pasteurised blackcurrant juice (dm-drogerie markt GmbH, Karlsruhe, Germany) with a chemical composition of: >0.5% (*w*/*w*) fat, 8.5% (*w*/*w*) carbohydrates, and 0.6% (*w*/*w*) protein (information obtained from the packaging). A water kefir starter culture (UNIBIOM s.r.o., Břeclav, Czech Republic) was utilised for the fermentation of the samples. The water kefir starter culture contained bacteria from the genera *Lactobacillus*, *Lactococcus*, *Leuconostoc*, *Acetobacter*, *Saccharomyces*, and *Kluyveromyces*, as reported by the producer.

### 2.2. Manufacture of the Fermented Beverage Models (Samples)

The fermented whey-based samples were manufactured using the back-sloping technique [26,27]. First, the inoculum was prepared by combining 0.50 ± 0.03 g of freeze-dried water kefir starter culture with 500 mL of distilled water. Additionally, 10% (*w*/*w*) of sucrose was also added in the inoculum. To create the active inoculum fermentation process was conducted at a temperature of 25 ± 2 °C for 72 h. Furthermore, whey powder was dispersed using the T 18 digital Ultra-Turrax (IKA-Werke GmbH, Staufen im Breisgau, Germany) in distilled water at a final concentration of 5% *w*/*w* (for 2 h at 20 ± 2 °C). Whey solution was then subjected to thermal treatment at 90 ± 1 °C for 10 ± 1 min and allowed to cool to 25 ± 2 °C. Thereafter, blackcurrant juice and whey were mixed, homogenised with a mixer device (ETA Dritto; ETA a.s., Prague, Czech Republic) for 3 min and then thermally treated (60 °C for 30 min; 2 °C/min) and left to cool (25 ± 2 °C). Subsequently, 10% (*w*/*w*) of the active inoculum was applied to all samples. Furthermore, a sample containing only whey was used as a control sample. Analyses were conducted at 4, 24, and 48 h of fermentation. A portion of 500 mL of each fermented beverage sample model was manufactured in total. Twenty-four batches of fermented whey-based beverage models were produced (*n* = 24), consisting of 4 different juice concentrations (including the control sample), 2 different hydrocolloid concentrations, and 3 repetitions. The samples were labelled as follows: F25_0 contained 0.25% *w*/*w* furcellaran, 0% *w*/*w* black currant juice and 100% *w*/*w* whey; F25_10 contained 0.25% *w*/*w* furcellaran, 10% *w*/*w* black currant juice and 90% *w*/*w* whey; F25_20 contained 0.25% *w*/*w* furcellaran, 20% *w*/*w* black currant juice and 80% *w*/*w* whey; F25_100 contained 0.25% *w*/*w* furcellaran, 100% *w*/*w* black currant juice and 0% *w*/*w* whey. F50_0 contained 0.50% *w*/*w* furcellaran, 0% *w*/*w* black currant juice and 100% *w*/*w* whey; F50_10 contained 0.50% *w*/*w* furcellaran, 10% *w*/*w* black currant juice and 90% *w*/*w* whey; F50_20 contained 0.50% *w*/*w* furcellaran, 20% *w*/*w* black currant juice and 80% *w*/*w* whey; F50_100 contained 0.50% *w*/*w* furcellaran, 100% *w*/*w* black currant juice and 0% *w*/*w* whey.

### 2.3. Psysicochemical Analysis and Sediment Content Determination

The pH values of the samples were measured at 20 ± 1 °C using a calibrated glass tip electrode of a pH meter (Edge; Hanna Instruments Czech s.r.o., Prague, Czech Republic) inserted directly into the samples. The total soluble solids (TSS; % *w*/*w*) of the samples were measured using a Kern OTSS 45BE digital refractometer (Kern & Sohn GmbH, Balingen, Germany). Measurements of TSS were conducted nine times (*n* = 9) at a temperature of 20 ± 2 °C. Ethanol concentration and density were measured using an Anton Paar Alcolyzer Plus and an Anton Paar DMA 4500 density meter (Anton Paar GmbH, Graz, Austria). Before measuring, the samples were centrifuged and degassed with an EBA 21 centrifuge (Hettich, Tuttlingen, Germany) at 6000 rpm for 10 min. Determination of CO_2_ content was performed during the fermentation period (48 h). After inoculation, 100 mL of each model was placed into a sealed plastic container. The sample was weighed, and the weight loss observed at the specified time intervals of 4, 24, and 48 h of fermentation was recorded.

Prior to the sediment content determination, the samples were centrifuged at 6000 rpm for 10 min using an EBA 21 centrifuge (Hettich, Tuttlingen, Germany). The supernatant was removed, and the sediment was weighed. The sediment content of the sample was calculated using Equation (1).

(1) $$S=\frac{m_{1}}{m_{0}}\cdot 100$$

where *S* is the sedimentation (rel.%), *m*_1_ is the weight of sediment (g), and *m*_0_ is the weight of sample (g).

All analyses, except for TSS, were conducted at least three times (*n* = 3).

### 2.4. Turbidity Analysis

Turbidity values of the samples were measured with an HI88173 turbidimeter (Hanna Instruments Czech s.r.o., Prague, Czech Republic). The instrument utilised an infrared LED lamp (860 nm) and followed the nephelometric method outlined in ISO 7027-1:2016. Turbidity observations are quantified in nephelometric turbidity units (NTU) [28,29]. Turbidity was assessed on the initial day of the experiment through three repetitions (*n* = 3), and the results are presented as the arithmetic mean of the obtained values.

### 2.5. Rheological Analysis

Rheological analysis was conducted using a HAAKE RheoStress 1 rheometer (Thermo Fisher Scientific Brno s.r.o., Brno, Czech Republic), which was equipped with a concentric cylinder geometry and a 2.1 mm gap was set. For each analysis, 1.0 mL of sample at a temperature of 20.0 ± 0.1 °C was utilised. The samples were measured throughout a range of shear rates from 0 to 500 s^−1^ to determine the steady-state rheological characteristics. Nonlinear regression analysis using the power-law (Equation (2)) and Herschel–Bulkley (Equation (3)) models were employed to analyse the flow curves. Each sample’s flow properties were assessed in a minimum of 9 times (*n* = 9).

(2) $$\tau {=K\dot{\gamma }}^{n}$$

where *τ* is the shear stress (Pa), *K* is the flow consistency index (Pa·s), $\dot{\gamma }$ is the shear rate (s^−1^), and *n* is the power-law index (dimensionless).

(3) $$\tau {=\tau_{0}+K\dot{\gamma }}^{n}$$

where *τ* is the shear stress (Pa), *τ*_0_ is the yield stress (Pa), *K* is the flow consistency index (Pa·s), $\dot{\gamma }$ is the shear rate (s^−1^), and *n* is the flow index (−).

### 2.6. Displacement Transmissibility Measurements

Dynamic mechanical properties of the investigated beverages were evaluated based on the displacement transmissibility *T_d_* (−), as expressed by the following Equation (4) [30,31]:

(4) $$T_{d}=\frac{y_{O}}{y_{I}}=\sqrt{\frac{k^{2}+{\left(c\omega \right)}^{2}}{{\left(k-m\omega^{2}\right)}^{2}+{\left(c\omega \right)}^{2}}}=\sqrt{\frac{1+{\left(2\zeta r\right)}^{2}}{{\left(1-r^{2}\right)}^{2}+{\left(2\zeta r\right)}^{2}}}$$

where *y* is the displacement amplitude on either the input (*I*) or output (*O*) sides of the tested sample (m), *k* is the sample stiffness (N/m), *c* is the viscous damping coefficient (N·s/m), *ω* is the circular frequency of oscillation (rad/s), *m* is the mass (kg), *ζ* is the damping ratio (−), and *r* is the frequency ratio (−).

In general, depending on the value of displacement transmissibility, mechanical vibrations can be classified into three different types: damped (*T_d_* < 1), undamped (*T_d_* = 1) and resonance (*T_d_* > 1) mechanical vibrations. Given the conditions *T_d_*/*dζ* = 0 in Equation (4), it is possible to determine the frequency ratio *r*_0_ at which the displacement transmissibility reaches its local extremum (i.e., its maximum value *T_dmax_*) at the first resonance frequency *f_R_*_1_ [30,32]:

(5) $$r_{0}=\frac{\sqrt{\sqrt{1+8\zeta^{2}}-1}}{2\zeta }$$

It is evident from Equation (5) that the maximum value of displacement transmissibility generally shifts to lower frequency ratios *r* with an increasing damping ratio *ζ* or decreasing mechanical stiffness *k* [33].

Experimental measurements of the displacement transmissibility of the tested beverage samples were performed using the method of harmonically excited mechanical vibrations within the frequency range of 2–200 Hz. The measuring apparatus consisted of a mini-shaker (BK 4810), a signal PULSE multi-analyser (BK 3560-B-030) and a power amplifier (BK 2706). In the case of the harmonically excited mechanical vibrations, Equation (5) can be expressed as follows:

(6) $$T_{d}=\frac{a_{O}}{a_{I}}$$

where *a* is the acceleration amplitude on either the input (*I*) or output (*O*) sides of the tested sample (m·s^−2^). The displacement transmissibility was determined using Equation (6) based on the acceleration amplitudes measured by the BK 4393 piezoelectric accelerometers *A_I_* and *A_O_* (Brüel & Kjær, Nærum, Denmark).

The frequency dependencies of the displacement transmissibility for the investigated beverage samples, each with a volume of 40 mL, were determined. These samples were properly vented and sealed in transparent polyethylene zip-lock bags. Subsequently, the sealed samples were placed in a metal vessel with dimensions of 60 mm × 60 mm × 20 mm (length × width × height) and were loaded with an inertial mass of 90 g on top of the harmonically loaded beverage samples. Each measurement was repeated 5 times under ambient conditions at a temperature of 22 ± 1 °C.

The displacement transmissibility analysis for mechanical vibration damping is usually applied to different types of solid materials, such as elastomers, polyurethane foams, cork, viscoelastic materials, and composites. As stated above, the ability of these materials to damp mechanical vibrations generally increases with an increasing damping ratio *ζ*. Additionally, this method has also been applied to investigate the mechanical vibration damping properties of some non-Newtonian fluids, such as plastic lubricants and processed cheese sauces [34,35]. Non-Newtonian fluids are also used as mechanical vibration dampers in various machines [36,37]. In these cases, the ability to damp mechanical vibrations during harmonic forced loading is related to the viscosity of non-Newtonian fluids. Generally, higher viscosity of fluids leads to higher internal friction during the propagation of mechanical waves through the fluids. This phenomenon results in higher dissipation of mechanical energy into heat and, consequently, a better ability to damp mechanical vibrations in fluids using the method of harmonically excited mechanical vibrations. This method can also be applied to compare the mechanical damping of non-Newtonian fluids. Therefore, displacement transmissibility analysis was used for the investigated fermented whey-based beverages which exhibited non-Newtonian pseudoplastic behaviour. It is a relatively quick, inexpensive, and non-destructive method for comparing the mechanical stiffness of materials, which are among its undeniable advantages.

### 2.7. Instrumental Analysis of Colour

The colour of the samples was determined using a HunterLab UltraScan Pro Colour spectrophotometer (Hunter Associates Laboratory, Inc., Reston, VA, USA). The analysis was conducted using the CIELAB colour space (also referred to as L*, a* and b*) under D65 normal daylight using a 10° angle. The parameter L* represents luminosity and ranges from 0 (black) to 100 (white). Parameter a* represents the colour spectrum from green (−) to red (+), while parameter b* represents the colour spectrum from blue (−) to yellow (+) [38]. The instrument was calibrated in the reflectance mode by removing specular reflection, utilizing white and black reference tiles. The hue angle (h* °) indicates the primary spectral component, like red, yellow, green, or blue, on a scale from 0° to 360°. An angle of 0° or 360° corresponds to a red hue, while angles of 90°, 180°, and 270° correspond to yellow, green, and blue hues, respectively. The variables a*, b*, and h* are used to provide a thorough description of colour and are determined using Equation (7).

(7) $${}^{\circ}h^{*}{=tan}^{-1}(b^{*}/a^{*})$$

The chroma (C*) value quantifies the intensity of a colour and is defined as follows (Equation (8)):

(8) $$C^{*}=({{a^{*}}^{2}+{b^{*}}^{2})}^{0.5}$$

### 2.8. Sensory Analysis

The samples were assessed based on sensory parameters including appearance, taste, aroma, overall rating, and off-flavours. Twelve expert assessors, consisting of 7 women and 5 men aged between 22 and 51 years old, participated in the sensory evaluation. The samples were presented in glass containers of 50 mL each, labelled with three-digit codes, and delivered in a random order at a consistent temperature of 20 ± 2 °C. The sensory evaluation occurred in a sensory analysis laboratory with individual sensory booths for each panellist, set up in accordance with the ISO 8589 standards [39]. Water was provided as a neutralizing agent. A 10 min break was implemented after each sample to avoid palate fatigue. The parameters of appearance, taste, and aroma were evaluated using a 5-point scale (1—excellent, 3—good, 5—unacceptable; each point on the scale was defined objectively with quality characteristics). The overall evaluation was based on a 5-point scale, with 1 representing extraordinarily good and 5 representing extraordinarily bad.

### 2.9. Statistical Analysis

The obtained data were presented as mean ± standard deviation and analysed using one-factor ANOVA and the Tukey’s post hoc test with 95% reliability. The sensory properties of the samples were evaluated using the Kruskal–Wallis and Wilcoxon tests. The tests were conducted using a significance level of 0.05. Statistical analyses were conducted using the Minitab^®^16 software (Minitab^®^, Ltd., Coventry, UK).

## 3. Results and Discussion

### 3.1. Psychicochemical and Turbidity Analyses

The results of the physicochemical analysis are shown in Table 1. The pH values of the fermented whey-based beverages were measured during fermentation. The results indicated that statistically significant differences between the samples (*p* < 0.05) occurred. The initial pH value of the samples (F25_0; F25_10; F25_20; F25_100) with the addition of 0.25% (*w*/*w*) furcellaran ranged from 2.77 to 6.18 and for the samples (F50_0; F50_10; F50_20; F50_100) with 0.50% (*w*/*w*) furcellaran from 2.77 to 6.14. The initial pH of the blackcurrant juice used in the study was slightly lower than that reported by Kelanne et al. [40] and Patelski et al. [41]. Additionally, the initial pH values of the whey were lower compared to the study by Levkov et al. [42]. During the fermentation, the pH levels decreased as a result of the production of organic acids, such as lactic acid [2]. Simultaneously, the low pH exhibited antibacterial properties and inhibited the growth of pathogenic microorganisms [43].

The TSS content was significantly higher in the samples containing 100% (*w*/*w*) blackcurrant juice and lowest in the samples containing 100% (*w*/*w*) whey. This suggested that the increasing amount of whey in the sample decreased the final TSS value of the samples (*p* < 0.05). On the other hand, no significant statistical differences were observed between samples with different furcellaran content (*p* ≥ 0.05). During fermentation, all samples showed a decrease in TSS values, which is due to the carbon conversion of fermentation metabolites [44]. Additionally, the decrease in TSS values is related to both ethanol formation and the CO_2_ content of the sample [45]. Furthermore, a decreasing trend in the density values of the tested samples was also recorded. In particular, the decrease in density, among other things, could serve as an indicator of the fermentation progress. The decrease is probably due to the formation of ethanol, CO_2_, organic acids and other sensory active substances [46,47]. All samples showed a statistically significant decrease in density (*p* < 0.05) during the whole fermentation time. The upper ethanol limit for an “alcohol-free beverage” in the majority of European countries is typically set at 0.5% (*v*/*v*) [48]. In general, the ethanol content of the samples increased with the increasing fermentation time (*p* < 0.05), ranging from 0.92 to 4.86% (*v*/*v*). The results of the ethanol content of the samples were in accordance with those previously presented in the studies of Kelanne et al. [40] and Randazzo et al. [49]. Furthermore, in the above-mentioned studies, the ethanol content of some samples fermented with water kefir cultures was higher than 4% *v*/*v*. Kefir and water kefir starter cultures include microorganisms responsible for ethanol production. The latter microorganisms are primarily yeasts, specifically *Saccharomyces cerevisiae*, which exhibit a strong fermentative metabolism. At the same time, certain genera of *Lactobacillus* contain the enzyme alcohol dehydrogenase and are therefore also capable of producing ethanol [49]. The most abundant microorganisms found in water kefir starter culture are yeasts *Saccharomyces bayanus*, *Saccharomyces cerevisiae*, lactic acid bacteria *Levilactobacillus brevis*, *Lentilactobacillus kefiri*, *Lentilactobacillus hilgardii*, *Lacticaseibacillus paracasei*, *Lactobacillus casei* subsp. *casei*, *Lactobacillus kefiranofaciens*, *Lactococcus cremoris*, *Lactococcus lactis* subsp. *Lactis* and, last but not least, the acetic acid bacteria *Acetobacter fabarum* and *Acetobacter orientalis*. Furthermore, it should be mentioned that the exact composition of microorganisms can vary depending on the source of the water kefir starter culture, the growth processes used during culture maintenance, and the geographical origin. [14,49]. There were statistically significant differences between the samples with different blackcurrant juice contents (*p* < 0.05); however, there were no significant differences between the samples with different furcellaran concentrations (*p* ≥ 0.05). A small amount of ethanol [<0.5% (*v*/*v*)] in a kefir product is important due to its contribution to the characteristic light alcoholic taste and yet simultaneously these beverages can be labelled as non-alcoholic. Additionally, the presence of CO_2_, primarily resulting from yeast fermentation, enhances the final product by imparting the desired notes and aroma [45,48].

Turbidity values are depicted in Table 1. The highest turbidity value was detected in the sample containing 100% (*w*/*w*) whey, regardless of the applied furcellaran content. On the other hand, the lowest turbidity was recorded in the samples containing 100% (*w*/*w*) fruit juice. The turbidity value decreased during fermentation in all samples (*p* < 0.05). In general, it could be stated that samples with higher whey content were cloudier.

**Figure 1:**
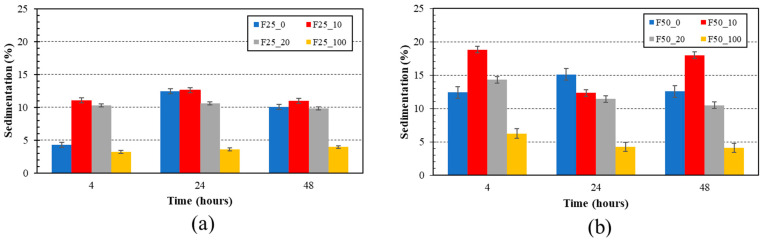
Effect of hydrocolloid concentration on sediment content in samples. Part (**a**) samples with 0.25% (*w*/*w*) of furcellaran, part (**b**) samples with 0.50% (*w*/*w*) of furcellaran. F25_0 contained 0.25% *w*/*w* furcellaran, 0% *w*/*w* black currant juice and 100% *w*/*w* whey; F25_10 contained 0.25% *w*/*w* furcellaran, 10% *w*/*w* black currant juice and 90% *w*/*w* whey; F25_20 contained 0.25% *w*/*w* furcellaran, 20% *w*/*w* black currant juice and 80% *w*/*w* whey; F25_100 contained 0.25% *w*/*w* furcellaran, 100% *w*/*w* black currant juice and 0% *w*/*w* whey. F50_0 contained 0.50% *w*/*w* furcellaran, 0% *w/w* black currant juice and 100% *w*/*w* whey; F50_10 contained 0.50% *w*/*w* furcellaran, 10% *w*/*w* black currant juice and 90% *w*/*w* whey; F50_20 contained 0.50% *w*/*w* furcellaran, 20% *w*/*w* black currant juice and 80% *w*/*w* whey; F50_100 contained 0.50% *w*/*w* furcellaran, 100% *w*/*w* black currant juice and 0% *w*/*w* whey.

Furthermore, the results of the sediment content determination are shown in Figure 1. Optimizing the amount of fining agent enhances the efficiency of filtration, increases colloidal stability, and promotes a denser sedimentation, resulting in minimised losses [50]. Sedimentation of the samples was probably caused by the reactions of whey proteins with the fruit component [20]. Higher concentrations of furcellaran decreased turbidity; however, from the data obtained, it can be reported that the fermented whey-based beverages with a higher furcellaran content showed a higher sediment content. There were statistically significant differences between the samples (*p* < 0.05). The samples F25_10 and F50_10 exhibited the highest sediment content following a 48 h fermentation period, with values of 10.99 (rel.%) and 17.98 (rel.%), respectively. Furthermore, based on the above-mentioned results, it could be stated that furcellaran is an effective fining agent. The efficiency of the fining process may be associated with the helical parameters (such as helical pitch and axial shift from the half-stagger position) and/or the charge density of the carrageenan samples [51].

**Figure 2:**
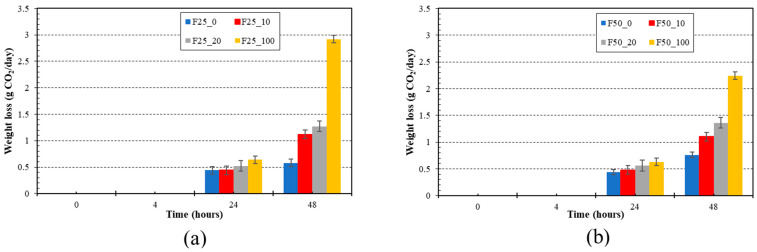
Fermentation kinetics. Evaluation of CO_2_ production of fermented whey-based beverage models. Part (**a**) samples with 0.25% (*w*/*w*) of furcellaran, part (**b**) samples with 0.50% (*w*/*w*) of furcellaran. F25_0 contained 0.25% *w*/*w* furcellaran, 0% *w*/*w* black currant juice and 100% *w*/*w* whey; F25_10 contained 0.25% *w*/*w* furcellaran, 10% *w*/*w* black currant juice and 90% *w*/*w* whey; F25_20 contained 0.25% *w*/*w* furcellaran, 20% *w*/*w* black currant juice and 80% *w*/*w* whey; F25_100 contained 0.25% *w*/*w* furcellaran, 100% *w*/*w* black currant juice and 0% *w*/*w* whey. F50_0 contained 0.50% *w*/*w* furcellaran, 0% *w*/*w* black currant juice and 100% *w*/*w* whey; F50_10 contained 0.50% *w*/*w* furcellaran, 10% *w*/*w* black currant juice and 90% *w*/*w* whey; F50_20 contained 0.50% *w*/*w* furcellaran, 20% *w*/*w* black currant juice and 80% *w*/*w* whey; F50_100 contained 0.50% *w*/*w* furcellaran, 100% *w*/*w* black currant juice and 0% *w*/*w* whey.

During the fermentation of the substrate with the water kefir starter culture, in addition to carbon dioxide, a number of other substances were produced, such as ethanol, lactic acid, mannitol, glycerol [52]. The samples were therefore subjected to weight loss due to the release of CO_2_ [53]. The weight loss of the fermented whey-based beverage model is shown in Figure 2. The formation of CO_2_ in the sample depends on the content of the fruit component in the sample (*p* < 0.05). The highest weight loss was obtained for the samples with 100% (*w*/*w*) blackcurrant juice content. Additionally, the maximum weight loss was observed after 48 h of fermentation for the F25_100 sample (2.28 ± 0.02 g CO_2_/day). The above-mentioned finding confirms that the presence of the fruit component is typical for the production of fermented beverages using water kefir culture [54]. The process of CO_2_ formation starts with the enzymatic conversion of sucrose into glucose by the enzyme glucose isomerase. Lactic acid bacteria utilize the resultant glucose for the production of lactic acid, whereas yeasts metabolize glucose to produce ethyl alcohol and CO_2_. The combination of low alcohol and carbon dioxide levels seen in beverages like kefir and water kefir produces a refreshing and yeast-like scent that consumers prefer in the finished product, similar to other carbonated beverages [55]. The CO_2_ level in water kefir beverages in this study corresponds to previous research conducted on various fermented matrices [45,55,56]. The weight loss results were in agreement with those of the ethanol content in the tested samples.

### 3.2. Rheological Analysis

To evaluate the flow curves of the whey-based fermented beverage models, the experimental data were fitted into two models (the power-law model and the Herschel–Bulkley model), from which the rheological parameters were obtained (Table 2). The Herschel–Bulkley model is frequently employed to characterize the flow behaviours of non-Newtonian fluids with yield stress. The *R*^2^ (coefficient of determination) for the power-law model was lower (0.93–0.96) than that for the Herschel–Bulkley model (0.99). The model fitness could be verified by the *R*^2^, as a value close to one indicating the most accurate fit of the model for calculating the correlation between shear stress and shear rate in the model samples [57]. According to the power-law model, all samples showed a pseudoplastic behaviour (*n* < 1) and the value of *n* decreased during fermentation. In contrast, the consistency index (*K*) exhibited oppositional behaviour compared to the flow behaviour index (*n*). The occurrence of pseudoplastic behaviour (also known as shear thinning) is attributed to the degradation of structural units, due to the hydrodynamic forces that are generated during the shearing process [58]. The furcellaran concentration showed no statistically significant effect (*p* ≥ 0.05) on the rheological properties of the model samples, whereas differences (*p* < 0.05) between samples with different concentrations of blackcurrant juice were detected.

**Figure 3:**
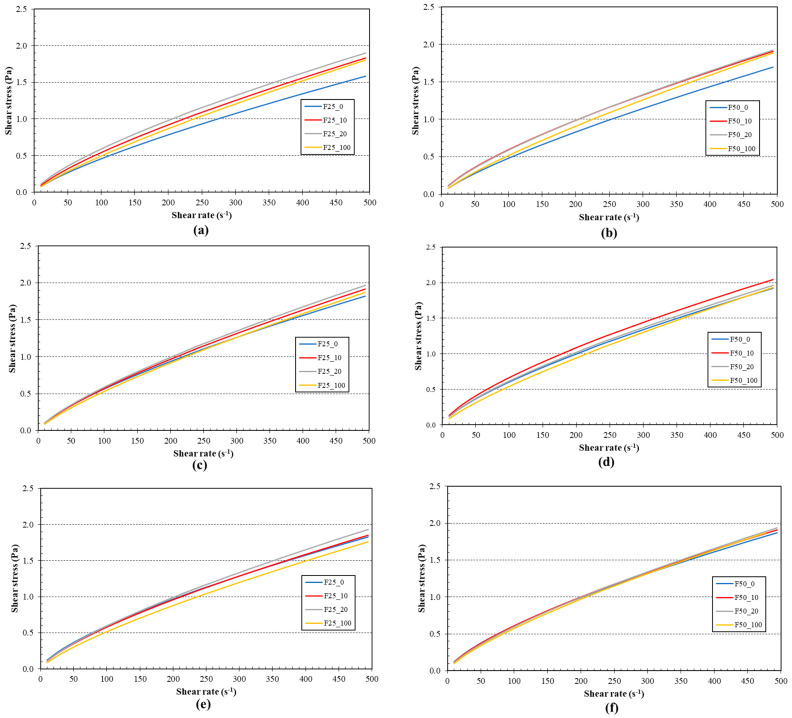
Flow curves (Power−law model) of the different models during the fermentation period (48 h); samples with 0.25% (*w*/*w*) of furcellaran 4 h (part (**a**)), 24 h (part (**c**)), 48 h (part (**e**)); model samples with 0.5% (*w*/*w*) of furcellaran 4 h (part (**b**)), 24 h (part (**d**)), 48 h (part (**f**)). F25_0 contained 0.25% *w*/*w* furcellaran, 0% *w*/*w* black currant juice and 100% *w*/*w* whey; F25_10 contained 0.25% *w*/*w* furcellaran, 10% *w*/*w* black currant juice and 90% *w*/*w* whey; F25_20 contained 0.25% *w*/*w* furcellaran, 20% *w*/*w* black currant juice and 80% *w*/*w* whey; F25_100 contained 0.25% *w*/*w* furcellaran, 100% *w*/*w* black currant juice and 0% *w*/*w* whey. F50_0 contained 0.50% *w*/*w* furcellaran, 0% *w*/*w* black currant juice and 100% *w*/*w* whey; F50_10 contained 0.50% *w/w* furcellaran, 10% *w*/*w* black currant juice and 90% *w*/*w* whey; F50_20 contained 0.50% *w*/*w* furcellaran, 20% *w*/*w* black currant juice and 80% *w*/*w* whey; F50_100 contained 0.50% *w*/*w* furcellaran, 100% *w*/*w* black currant juice and 0% *w*/*w* whey.

Viscosity is a crucial characteristic in milk beverages that may affect the flow characteristics and is also associated with the mouthfeel. Figure 3 shows the flow curves of the model samples as affected by fermentation time (48 h). It can be reported that changes in the flow curves occurred during fermentation. In particular, the shear stress increased with increasing shear rate; thus, all the models’ samples exhibited a non-Newtonian pseudoplastic behaviour. At the beginning of fermentation (after 4 h of fermentation), the sample containing only 100% (*w*/*w*) whey showed the lowest viscosity for both furcellaran concentrations (part a and b). With the increasing level of blackcurrant juice, the viscosity of the model samples also increased.

### 3.3. Mechanical Vibration Damping Analysis

**Figure 4:**
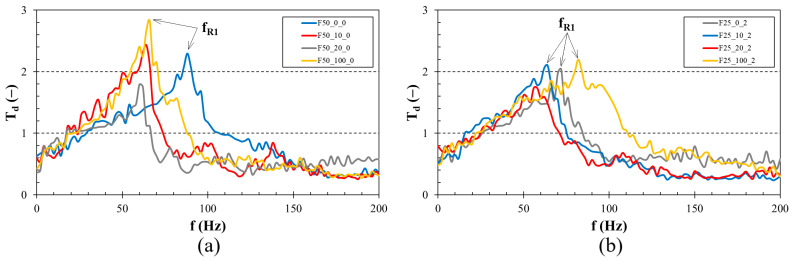
Effect of blackcurrant juice concentration on displacement transmissibility in relation to the frequency: (**a**) 0.50% (*w*/*w*) concentration of furcellaran, 4 h of fermentation, (**b**) 0.25% (*w*/*w*) concentration of furcellaran; 48 h of fermentation. *f_R_*_1_—first resonance frequency (*f_R_*_1_ ≈ *T_dmax_*).

**Figure 5:**
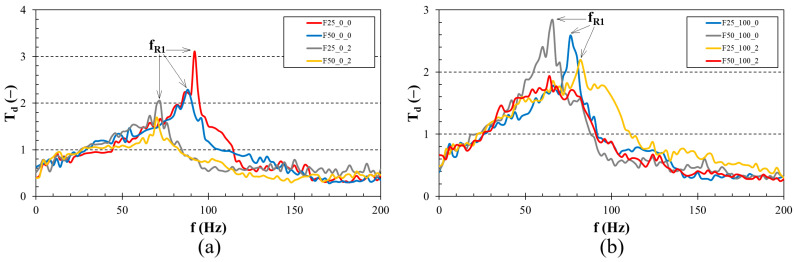
Effect of furcellaran concentration and fermentation time on displacement transmissibility in relation to the frequency: (**a**) 0% (*w*/*w*) concentration of blackcurrant juice, (**b**) 100% (*w*/*w*) concentration of blackcurrant juice. *f_R_*_1_—first resonance frequency (*f_R_*_1_ ≈ *T_dmax_*).

Figure 4 and Figure 5 show examples of the frequency dependencies of the displacement transmissibility of the investigated beverage samples. It is evident that resonant mechanical oscillations occur at low excitation frequencies (i.e., at *T_d_* > 1). Conversely, at higher excitation frequencies, there is a damped mechanical oscillation (i.e., when *T_d_* < 1), which is influenced by the concentration of furcellaran and blackcurrant juice as well as the fermentation time.

The influence of blackcurrant juice concentration on the displacement transmissibility is depicted in Figure 4a,b. Figure 4a specifically examines the frequency dependencies of *T_d_* for samples with a 0.50% (*w*/*w*) furcellaran concentration after 4 h of fermentation. The findings reveal that the sample with 0% (*w*/*w*) blackcurrant juice concentration exhibited the lowest vibration damping properties, demonstrated by a higher first resonance frequency (i.e., *f_R_*_1_ = 87 Hz) compared to other beverage samples. Consequently, this sample is characterised by low viscous friction. Conversely, the highest ability to damp mechanical vibrations (i.e., the highest viscous friction) was observed for the beverage sample with a 20% (*w*/*w*) blackcurrant juice concentration, which exhibited a first resonance frequency of 61 Hz. The effect of blackcurrant juice concentration on the displacement transmissibility after 48 h of fermentation for samples with a 0.25% (*w*/*w*) furcellaran concentration is depicted in Figure 4b. Here, the highest first resonance frequency (i.e., *f_R_*_1_ = 82 Hz) and thus the lowest ability to damp mechanical vibration was found for the sample containing 100% (*w*/*w*) blackcurrant juice. In contrast, the highest vibration damping properties (i.e., *f_R_*_1_ = 58 Hz) were observed for the beverage sample with a 20% (*w*/*w*) blackcurrant juice concentration.

Figure 5a,b demonstrate the impact of furcellaran concentration and fermentation time on the displacement transmissibility. A higher concentration of furcellaran in the beverage samples consistently resulted in lower first resonance frequency values. Consequently, increased furcellaran concentration enhanced the samples’ viscosity, thereby improving their capacity to damp mechanical vibrations. As shown in Table 3, the effect of the furcellaran concentration on the first resonance frequency was the same for all studied beverage samples. Similarly, longer fermentation times generally led to lower first resonance frequencies. Nevertheless, for the samples with 0.25% (*w*/*w*) furcellaran and 100% (*w*/*w*) blackcurrant juice, the effect of fermentation time was reversed. Specifically, the first resonance frequency increased from 77 to 82 Hz as the fermentation time extended from 4 to 48 h. However, Table 3 indicates that, regardless of furcellaran and blackcurrant juice concentrations, the lowest first resonance frequencies and the highest viscous friction were found in beverage samples fermented for 24 h. Table 3 clearly shows that the sample with 0.50% (*w*/*w*) furcellaran and 10% (*w*/*w*) blackcurrant juice, fermented for 24 h, exhibited the highest viscous friction (i.e., *f_R_*_1_ = 50 Hz). Conversely, the sample with 0.25% (*w*/*w*) furcellaran and 0% (*w*/*w*) blackcurrant juice, fermented for 4 h, exhibited the lowest ability (i.e., *f_R_*_1_ = 92 Hz) to damp mechanical vibrations.

It can be concluded that the findings mentioned above align excellently with the rheological analysis, which showed that a higher viscosity in the beverage samples generally led to increased viscous friction and, consequently, an enhanced ability to damp mechanical vibrations and a higher conversion of mechanical energy into heat during forced oscillations. For this reason, beverage samples with high viscosity are characterised by an increased damping ratio, *ζ*. This phenomenon corresponds to Equation (5), which demonstrates that the frequency ratio *r*_0_ decreases as the damping ratio *ζ* increases, resulting in a lower first resonance frequency *f_R_*_1_.

### 3.4. Instrumental Colour Analysis

The values of the instrumental analysis of colour are shown in Table 4. In general, the blackcurrant juice content and fermentation time affected the resulting colorimetric parameters (*p* < 0.05), whereas the furcellaran concentration did not affect these parameters (*p* > 0.05). As the blackcurrant juice concentration increased, the L* and b* values decreased, whereas the a* value increased. The parameters a* and b* assumed positive values throughout the whole fermentation period; therefore, the model samples can be described as red with a yellow tint and yellow with a red tint, respectively, depending on the whey and blackcurrant juice contents. The highest value of chroma (colour saturation) was detected in the samples F25_10 and F50_10 containing 10% (*w*/*w*) of blackcurrant juice. The lowest hue angle value was observed in the samples containing 100% (*w*/*w*) blackcurrant juice, indicating a greater proximity to the red colour. On the other hand, samples containing 100% (*w*/*w*) whey showed more yellow tones, as the h* values were close to 90°. The colour parameters of the models’ samples changed during fermentation, which corresponds with the results presented by Şafak et al. [44].

### 3.5. Sensory Analysis

The results of the sensory analysis are shown in Table 5. The fermented whey-based beverage models were evaluated for appearance, taste, aroma, off-flavour and overall rating. Based on the sensory analysis, it was found that both blackcurrant juice and furcellaran content affected all organoleptic properties of the samples (*p* < 0.05). The evaluators rated the appearance of the samples with 100% (*w*/*w*) whey and 10% (*w*/*w*) blackcurrant juice as the worst. The taste of the samples was rated as having bad marks, mainly due to the high acidity of the samples. The models with a lower juice content, respectively, to whey, also had undesirable off-flavours. The best sample in overall rating was sample F50_20. Many studies report that the sensory properties of water kefir beverages vary according to the type and amount of water kefir culture, the fermentation and storage temperature, the type of fruit or vegetable used and the water-soluble matter content [45,49,56,59].

## 4. Conclusions

In this study, model samples (fermented with water kefir starter culture) with different proportions of blackcurrant juice/whey and furcellaran were produced. The tested physicochemical parameters and turbidity of the samples were affected by the blackcurrant juice level and the fermentation time. In particular, the pH value decreased with increasing blackcurrant juice content; on the other hand, the ethanol content increased. The sediment content was higher in the samples with 0.50% (*w*/*w*) furcellaran addition, depicting its excellent fining properties. From the results of the rheological analysis, all samples can be considered as non-Newtonian pseudoplastic fluids in which the viscosity changed during fermentation. Colorimetric analysis showed that all samples reported different colorimetric parameters during fermentation. Furthermore, the rheological findings were confirmed using a non-destructive mechanical vibration damping method, where an increase in viscosity caused a shift in the first resonance frequency peak position towards lower excitation frequencies. It can be concluded that the displacement transmissibility method can be used to compare the mechanical damping not only of solid materials but also of non-Newtonian fluids. Combining whey and blackcurrant juice may offer improved flavour and palatability. The blackcurrant tart and fruity flavour can improve the organoleptic profile of whey-based beverages. Based on the result of the sensory analysis, some of the model samples were not well accepted by the evaluators, mainly due to unsatisfactory taste and appearance. However, the sample F50_20 containing 0.50% (*w*/*w*) furcellaran and 20% (*w*/*w*) blackcurrant juice was evaluated as the best in terms of sensory properties. Whey is increasingly used as a raw material for the production of various types of food products. Due to its organoleptic properties, it is often combined with fruit/vegetable juices. Additionally, the use of blackcurrant juice may appear to be advantageous due to its high content of substances with antioxidant activity. Hence, a further follow-up study could be an investigation of the antioxidant activity of the model samples and will be used for a future article dedicated to the functional properties of the products.

## Figures and Tables

**Table 1:** Physicochemical parameters of the evaluated fermented whey beverages enriched with blackcurrant juice *, **, ***.

Samples *	Time (h)	pH	TSS ^1^ (°Bx)	Density (kg·m^−3^)	Ethanol (% *v*/*v*)	Turbidity (NTU)
F25_0	4	6.18 ± 0.03 ^a,A^	5.13 ± 0.05 ^a,A^	1.021 ± 0.001 ^a,A^	0.06 ± 0.02 ^a,A^	824 ± 1 ^a,A^
F25_10		3.86 ± 0.02 ^b,A^	5.53 ± 0.09 ^b,A^	1.024 ± 0.001 ^b,A^	0.02 ± 0.01 ^b,A^	762 ± 1 ^b,A^
F25_20		3.41 ± 0.01 ^c,A^	6.83 ± 0.05 ^c,A^	1.029 ± 0.002 ^c,A^	0.03 ± 0.01 ^b,A^	682 ± 1 ^c,A^
F25_100		2.77 ± 0.01 ^d,A^	15.43 ± 0.05 ^d,A^	1.064 ± 0.002 ^d,A^	0.03 ± 0.01 ^b,A^	564 ± 4 ^d,A^
F50_0		6.14 ± 0.03 ^a,A^	5.33 ± 0.05 ^e,A^	1.021 ± 0.001 ^a,A^	0.06 ± 0.02 ^a,A^	855 ± 1 ^e,A^
F50_10		3.85 ± 0.02 ^b,A^	5.97 ± 0.05 ^f,A^	1.025 ± 0.002 ^b,A^	0.01 ± 0.01 ^b,A^	753 ± 2 ^f,A^
F50_20		3.40 ± 0.03 ^c,A^	6.73 ± 0.05 ^g,A^	1.029 ± 0.001 ^c,A^	0.02 ± 0.01 ^b,A^	672 ± 1 ^g,A^
F50_100		2.77 ± 0.01 ^d,A^	15.63 ± 0.05 ^h,A^	1.066 ± 0.002 ^d,A^	0.04 ± 0.02 ^b,A^	563 ± 4 ^h,A^
F25_0	24	4.25 ± 0.02 ^a,B^	3.57 ± 0.05 ^a,B^	1.015 ± 0.002 ^a,B^	0.79 ± 0.02 ^a,B^	811 ± 1 ^a,B^
F25_10		4.50 ± 0.03 ^b,B^	4.87 ± 0.05 ^b,B^	1.016 ± 0.001 ^b,B^	1.05 ± 0.01 ^b,B^	714 ± 1 ^b,B^
F25_20		3.36 ± 0.01 ^c,B^	5.77 ± 0.09 ^c,B^	1.020 ± 0.002 ^c,B^	1.18 ± 0.02 ^c,B^	676 ± 2 ^c,B^
F25_100		2.74 ± 0.01 ^d,B^	14.77 ± 0.05 ^d,B^	1.058 ± 0.003 ^d,B^	1.31 ± 0.02 ^d,B^	427 ± 1 ^d,B^
F50_0		4.28 ± 0.02 ^a,B^	3.73 ± 0.05 ^e,B^	1.015 ± 0.002 ^a,B^	0.79 ± 0.01 ^a,B^	802 ± 2 ^e,B^
F50_10		4.48 ± 0.02 ^b,B^	5.10 ± 0.08 ^f,B^	1.017 ± 0.001 ^b,B^	1.05 ± 0.01 ^b,B^	750 ± 1 ^f,A^
F50_20		3.36 ± 0.03 ^c,A^	5.83 ± 0.12 ^g,B^	1.020 ± 0.001 ^c,B^	1.18 ± 0.02 ^c,B^	670 ± 2 ^g,A^
F50_100		2.77 ± 0.03 ^d,A^	15.17 ± 0.09 ^h,B^	1.054 ± 0.002 ^d,B^	1.32 ± 0.02 ^d,B^	557 ± 3 ^h,A^
F25_0	48	3.83 ± 0.02 ^a,C^	2.73 ± 0.05 ^a,C^	1.014 ± 0.002 ^a,C^	0.92 ± 0.02 ^a,C^	744 ± 1 ^a,C^
F25_10		4.08 ± 0.03 ^b,C^	3.73 ± 0.09 ^b,C^	1.015 ± 0.001 ^b,C^	1.18 ± 0.01 ^b,C^	681 ± 2 ^b,C^
F25_20		3.35 ± 0.02 ^c,B^	4.30 ± 0.14 ^c,C^	1.017 ± 0.001 ^c,C^	1.58 ± 0.01 ^c,C^	635 ± 2 ^c,C^
F25_100		2.73 ± 0.01 ^d,B^	9.60 ± 0.02 ^d,C^	1.031 ± 0.001 ^d,C^	4.86 ± 0.01 ^d,C^	325 ± 1 ^d,C^
F50_0		3.85 ± 0.01 ^a,C^	2.87 ± 0.05 ^e,C^	1.014 ± 0.002 ^a,B^	0.92 ± 0.01 ^a,C^	763 ± 2 ^e,C^
F50_10		4.13 ± 0.02 ^b,C^	3.27 ± 0.05 ^f,C^	1.015 ± 0.002 ^b,B^	1.31 ± 0.02 ^d,C^	697 ± 2 ^f,C^
F50_20		3.35 ± 0.01 ^c,A^	3.90 ± 0.08 ^g,C^	1.016 ± 0.001 ^c,C^	1.71 ± 0.01 ^e,C^	620 ± 3 ^g,C^
F50_100		2.75 ± 0.02 ^d,A^	10.43 ± 0.05 ^h,C^	1.029 ± 0.002 ^d,C^	4.59 ± 0.01 ^f,C^	314 ± 3 ^h,C^

* Values are presented as the mean ± SD; ** F25_0 contained 0.25% *w*/*w* furcellaran, 0% *w*/*w* black currant juice and 100% *w*/*w* whey; F25_10 contained 0.25% *w*/*w* furcellaran, 10% *w*/*w* black currant juice and 90% *w*/*w* whey; F25_20 contained 0.25% *w*/*w* furcellaran, 20% *w*/*w* black currant juice and 80% *w*/*w* whey; F25_100 contained 0.25% *w*/*w* furcellaran, 100% *w*/*w* black currant juice and 0% *w*/*w* whey. F50_0 contained 0.50% *w*/*w* furcellaran, 0% *w*/*w* black currant juice and 100% *w*/*w* whey; F50_10 contained 0.50% *w*/*w* furcellaran, 10% *w*/*w* black currant juice and 90% *w*/*w* whey; F50_20 contained 0.50% *w*/*w* furcellaran, 20% *w*/*w* black currant juice and 80% *w*/*w* whey; F50_100 contained 0.50% *w*/*w* furcellaran, 100% *w*/*w* black currant juice and 0% *w*/*w* whey. *** Mean values within a column (the difference between black currant juice level, comparing the same fermentation time) followed by different superscript letters statistically differ (*p* < 0.05). Mean values within a column (the difference between fermentation time, comparing the same black currant juice level and furcellaran concentration) followed by different uppercase letters differ (*p* < 0.05). ^1^ TSS: total soluble solids.

**Table 2:** Rheological parameters of the tested fermented whey-based beverages obtained by the power-law and the Herschel–Bulkley models *, **, ***.

		Power-Law Model			Herschel–Bulkley Model			
Samples *	Time (h)	*K* ^1^ (Pa·s)	*N* ^1^ (−)	*R*^2^ (−)	*τ*_0_ ^1^ (Pa)	*K* ^1^ (Pa·s)	*N* ^1^ (−)	*R*^2^ (−)
F25_0	4	0.0127 ^a,A^	0.778 ^a,A^	0.93	0.1697 ^a,A^	0.001 ^a,A^	1.601 ^a,A^	0.99
F25_10		0.0163 ^b,A^	0.761 ^b,A^	0.94	0.2367 ^b,A^	0.001 ^a,A^	1.716 ^b,A^	0.99
F25_20		0.0205 ^c,A^	0.732 ^c,A^	0.94	0.2678 ^c,A^	0.001 ^a,A^	1.682 ^c,A^	0.99
F25_100		0.0120 ^d,A^	0.808 ^d,A^	0.94	0.1886 ^d,A^	0.001 ^a,A^	1.681 ^d,A^	0.99
F50_0		0.0126 ^a,A^	0.791 ^e,A^	0.93	0.1861 ^e,A^	0.001 ^a,A^	1.664 ^e,A^	0.99
F50_10		0.0209 ^e,A^	0.730 ^f,A^	0.94	0.2827 ^f,A^	0.001 ^a,A^	1.726 ^f,A^	0.99
F50_20		0.0196 ^f,A^	0.740 ^g,A^	0.94	0.2731 ^g,A^	0.001 ^a,A^	1.734 ^g,A^	0.99
F50_100		0.0126 ^g,A^	0.807 ^h,A^	0.93	0.2002 ^h,A^	0.001 ^a,A^	1.701 ^h,A^	0.99
F25_0	24	0.0192 ^a,B^	0.734 ^a,B^	0.93	0.2499 ^a,B^	0.001 ^a,A^	1.680 ^a,B^	0.99
F25_10		0.0178 ^b,B^	0.754 ^b,B^	0.94	0.2450 ^b,B^	0.001 ^a,A^	1.663 ^b,B^	0.99
F25_20		0.0180 ^b,B^	0.757 ^c,B^	0.95	0.2506 ^c,B^	0.001 ^a,A^	1.619 ^c,B^	0.99
F25_100		0.0140 ^d,B^	0.790 ^d,B^	0.94	0.1966 ^d,B^	0.001 ^a,A^	1.604 ^d,B^	0.99
F50_0		0.0214 ^e,B^	0.725 ^e,B^	0.94	0.2888 ^e,B^	0.001 ^a,A^	1.726 ^e,B^	0.99
F50_10		0.0263 ^f,B^	0.702 ^f,B^	0.96	0.2865 ^f,B^	0.001 ^a,A^	1.438 ^f,B^	0.99
F50_20		0.0223 ^g,B^	0.722 ^g,B^	0.95	0.2846 ^g,B^	0.001 ^a,A^	1.611 ^g,B^	0.99
F50_100		0.0141 ^h,B^	0.794 ^h,B^	0.94	0.2112 ^h,B^	0.001 ^a,A^	1.670 ^h,B^	0.99
F25_0	48	0.0234 ^a,C^	0.705 ^a,C^	0.94	0.2887 ^a,C^	0.001 ^a,A^	1.723 ^a,C^	0.99
F25_10		0.0201 ^b,C^	0.729 ^b,C^	0.94	0.2744 ^b,C^	0.001 ^a,A^	1.730 ^b,C^	0.99
F25_20		0.0199 ^c,C^	0.740 ^c,C^	0.94	0.2503 ^c,C^	0.001 ^a,A^	1.602 ^c,C^	0.99
F25_100		0.0151 ^d,C^	0.768 ^d,C^	0.93	0.2039 ^d,C^	0.001 ^a,A^	1.642 ^d,C^	0.99
F50_0		0.0241 ^e,C^	0.701 ^e,C^	0.94	0.3034 ^e,C^	0.001 ^a,A^	1.730 ^e,C^	0.99
F50_10		0.0232 ^f,C^	0.713 ^f,C^	0.94	0.3045 ^f,C^	0.001 ^a,A^	1.735 ^f,C^	0.99
F50_20		0.0201 ^g,C^	0.737 ^g,C^	0.94	0.2746 ^g,C^	0.001 ^a,A^	1.684 ^g,C^	0.99
F50_100		0.0180 ^h,C^	0.753 ^h,C^	0.95	0.2420 ^h,C^	0.001 ^a,A^	1.579 ^h,C^	0.99

* Values are presented as the mean ± SD; ** F25_0 contained 0.25% *w*/*w* furcellaran, 0% *w*/*w* black currant juice and 100% *w*/*w* whey; F25_10 contained 0.25% *w*/*w* furcellaran, 10% *w*/*w* black currant juice and 90% *w*/*w* whey; F25_20 contained 0.25% *w*/*w* furcellaran, 20% *w*/*w* black currant juice and 80% *w*/*w* whey; F25_100 contained 0.25% *w*/*w* furcellaran, 100% *w*/*w* black currant juice and 0% *w*/*w* whey. F50_0 contained 0.50% *w*/*w* furcellaran, 0% *w*/*w* black currant juice and 100% *w*/*w* whey; F50_10 contained 0.50% *w*/*w* furcellaran, 10% *w*/*w* black currant juice and 90% *w*/*w* whey; F50_20 contained 0.50% *w*/*w* furcellaran, 20% *w*/*w* black currant juice and 80% *w*/*w* whey; F50_100 contained 0.50% *w*/*w* furcellaran, 100% *w*/*w* black currant juice and 0% *w*/*w* whey. *** Mean values within a column (the difference between blackcurrant juice level, comparing the same fermentation time) followed by different superscript letters statistically differ (*p* < 0.05). Mean values within a column (the difference between fermentation time, comparing the same blackcurrant juice level and furcellaran concentration) followed by different uppercase letters differ (*p* < 0.05). ^1^ *K*: consistency index; *n*: flow behaviour index; *τ*_0_: yield value.

**Table 3:** First resonance frequency *f_R_*_1_ (Hz) of the investigated beverage samples with a volume of 40 mL sealed in transparent polyethylene zip-lock bags and loaded with an inertial mass of 90 g *, **, ***.

Samples **	Time (h)	First Resonance Frequency (Hz)
F25_0	4	92 ± 8 ^a,A^
F25_10		70 ± 5 ^b,A^
F25_20		69 ± 6 ^b,A^
F25_100		77 ± 7 ^b,A^
F50_0		87 ± 7 ^a,A^
F50_10		66 ± 5 ^b,A^
F50_20		61 ± 3 ^b,A^
F50_100		67 ± 4 ^b,A^
F25_0	24	77 ± 4 ^a,B^
F25_10		64 ± 3 ^b,B^
F25_20		52 ± 3 ^c,B^
F25_100		70 ± 5 ^a,B^
F50_0		61 ± 6 ^a,B^
F50_10		50 ± 3 ^b,B^
F50_20		53 ± 4 ^b,B^
F50_100		57 ± 4 ^b,B^
F25_0	48	72 ± 5 ^a,B^
F25_10		66 ± 6 ^a,B^
F25_20		58 ± 5 ^b,B^
F25_100		82 ± 6 ^c,B^
F50_0		71 ± 6 ^a,B^
F50_10		65 ± 4 ^a,B^
F50_20		56 ± 5 ^b,B^
F50_100		63 ± 5 ^a,B^

* Results are expressed as mean value ± SD. Mean value within the columns followed by different superscripts statistically differed (*p* < 0.05). ** F25_0 contained 0.25% *w*/*w* furcellaran, 0% *w*/*w* black currant juice and 100% *w*/*w* whey; F25_10 contained 0.25% *w*/*w* furcellaran, 10% *w*/*w* black currant juice and 90% *w*/*w* whey; F25_20 contained 0.25% *w*/*w* furcellaran, 20% *w*/*w* black currant juice and 80% *w*/*w* whey; F25_100 contained 0.25% *w*/*w* furcellaran, 100% *w*/*w* black currant juice and 0% *w*/*w* whey. F50_0 contained 0.50% *w*/*w* furcellaran, 0% *w*/*w* black currant juice and 100% *w*/*w* whey; F50_10 contained 0.50% *w*/*w* furcellaran, 10% *w*/*w* black currant juice and 90% *w*/*w* whey; F50_20 contained 0.50% *w*/*w* furcellaran, 20% *w*/*w* black currant juice and 80% *w*/*w* whey; F50_100 contained 0.50% *w*/*w* furcellaran, 100% *w*/*w* black currant juice and 0% *w*/*w* whey. *** Mean values within a column (the difference between blackcurrant juice level, comparing the same fermentation time) followed by different superscript letters statistically differ (*p* < 0.05). Mean values within a column (the difference between fermentation time, comparing the same blackcurrant juice level and furcellaran concentration) followed by different uppercase letters differ (*p* < 0.05).

**Table 4:** Colorimetric parameters (L* luminosity; a* from green (−) to red (+); b* from blue (−) to yellow (+), chroma, hue angle) results of the model samples *, **, ***.

Sample	Time (h)	L* (−)	a* (−)	b* (−)	C* (−)	*h* (°)
F25_0	4	69.73 ± 0.03 ^a,A^	0.97 ± 0.06 ^a,A^	16.72 ± 0.11 ^a,A^	16.75 ± 0.11 ^a,A^	86.69 ± 0.19 ^a,A^
F25_10		21.15 ± 1.30 ^b,A^	29.17 ± 0.70 ^b,A^	26.45 ± 0.39 ^b,A^	39.38 ± 0.30 ^b,A^	42.19 ± 0.38 ^b,A^
F25_20		7.72 ± 0.38 ^c,A^	30.89 ± 0.51 ^c,A^	12.27 ± 0.65 ^c,A^	33.24 ± 0.71 ^c,A^	21.66 ± 0.72 ^c,A^
F25_100		2.86 ± 0.01 ^d,A^	18.99 ± 0.06 ^d,A^	4.93 ± 0.02 ^d,A^	19.62 ± 0.07 ^d,A^	14.54 ± 0.01 ^d,A^
F50_0		69.37 ± 0.15 ^e,A^	1.42 ± 0.05 ^e,A^	17.97 ± 0.15 ^e,A^	18.02 ± 0.15 ^e,A^	85.48 ± 0.13 ^e,A^
F50_10		21.20 ± 0.51 ^f,A^	29.85 ± 0.09 ^f,A^	26.65 ± 0.27 ^b,A^	40.01 ± 0.22 ^f,A^	41.76 ± 0.25 ^f,A^
F50_20		7.80 ± 0.65 ^g,A^	31.49 ± 0.67 ^g,A^	13.44 ± 1.12 ^c,A^	34.24 ± 1.05 ^c,A^	23.08 ± 1.31 ^g,A^
F50_100		2.91 ± 0.05 ^h,A^	19.58 ± 0.31 ^h,A^	5.01 ± 0.08 ^f,A^	20.21 ± 0.32 ^g, A^	14.35 ± 0.02 ^h,A^
F25_0	24	66.53 ± 0.07 ^a,B^	0.95 ± 0.23 ^a,B^	13.28 ± 0.92 ^a,B^	13.31 ± 0.93 ^a,B^	85.92 ± 0.86 ^a,B^
F25_10		19.35 ± 0.63 ^b,B^	21.99 ± 0.11 ^b,B^	25.42 ± 0.29 ^b,B^	33.61 ± 0.16 ^b,B^	49.13 ± 0.45 ^b,B^
F25_20		7.44 ± 0.93 ^c,A^	30.79 ± 1.12 ^c,B^	12.83 ± 1.60 ^c,B^	33.36 ± 0.16 ^c,B^	22.55 ± 1.80 ^c,B^
F25_100		2.57 ± 0.09 ^d,B^	16.99 ± 0.57 ^d,B^	4.38 ± 0.38 ^d,B^	17.54 ± 0.59 ^d,B^	14.47 ± 0.03 ^d,B^
F50_0		67.70 ± 0.78 ^e,B^	1.17 ± 0.09 ^e,B^	13.97 ± 0.41 ^a,B^	14.02 ± 0.41 ^a,B^	85.24 ± 0.29 ^a,B^
F50_10		26.16 ± 0.81 ^f,B^	20.54 ± 0.39 ^f,B^	24.83 ± 0.25 ^e,B^	32.22 ± 0.43 ^e,B^	50.41 ± 0.28 ^e,B^
F50_20		7.88 ± 1.23 ^g,A^	31.15 ± 1.29 ^c,B^	13.58 ± 2.11 ^c,B^	34.01 ± 1.02 ^c,B^	23.44 ± 2.37 ^c,B^
F50_100		2.09 ± 0.25 ^h,B^	14.17 ± 1.66 ^d,B^	3.61 ± 0.43 ^f,B^	14.63 ± 1.72 ^f,B^	14.28 ± 0.05 ^f,B^
F25_0	48	65.37 ± 0.49 ^a,C^	1.06 ± 0.05 ^a,C^	13.18 ± 0.26 ^a,C^	13.22 ± 0.25 ^a,C^	85.38 ± 0.29 ^a,C^
F25_10		19.11 ± 0.33 ^b,C^	24.00 ± 0.22 ^b,C^	25.81 ± 0.59 ^b,C^	35.25 ± 0.28 ^b,C^	47.08 ± 0.91 ^b,C^
F25_20		7.49 ± 0.95 ^c,C^	30.67 ± 1.14 ^c,C^	12.90 ± 1.64 ^c,C^	33.29 ± 1.68 ^c,C^	22.74 ± 1.86 ^c,C^
F25_100		0.84 ± 0.09 ^d,C^	5.69 ± 0.57 ^d,C^	1.44 ± 0.15 ^d,C^	5.87 ± 0.60 ^d,C^	14.20 ± 0.06 ^d,C^
F50_0		62.61 ± 1.33 ^e,C^	1.08 ± 0.12 ^a,C^	13.49 ± 0.14 ^a,C^	13.53 ± 0.13 ^a,C^	85.42 ± 0.57 ^a,C^
F50_10		23.78 ± 1.21 ^f,C^	22.66 ± 0.16 ^e,C^	25.97 ± 0.29 ^b,C^	34.47 ± 0.32 ^b,C^	48.89 ± 0.12 ^e,C^
F50_20		6.21 ± 0.91 ^g,C^	28.81 ± 1.60 ^c,C^	10.72 ± 1.57 ^c,C^	30.75 ± 2.05 ^e,C^	20.32 ± 1.67 ^c,C^
F50_100		0.76 ± 0.08 ^h,C^	5.20 ± 0.54 ^d,C^	1.31 ± 0.14 ^d,C^	5.36 ± 0.56 ^d,C^	14.10 ± 0.03 ^d,C^

* Values are presented as the mean ± SD; ** F25_0 contained 0.25% *w*/*w* furcellaran, 0% *w*/*w* black currant juice and 100% *w*/*w* whey; F25_10 contained 0.25% *w*/*w* furcellaran, 10% *w*/*w* black currant juice and 90% *w*/*w* whey; F25_20 contained 0.25% *w*/*w* furcellaran, 20% *w*/*w* black currant juice and 80% *w*/*w* whey; F25_100 contained 0.25% *w*/*w* furcellaran, 100% *w*/*w* black currant juice and 0% *w/w* whey. F50_0 contained 0.50% *w*/*w* furcellaran, 0% *w*/*w* black currant juice and 100% *w*/*w* whey; F50_10 contained 0.50% *w*/*w* furcellaran, 10% *w*/*w* black currant juice and 90% *w*/*w* whey; F50_20 contained 0.50% *w*/*w* furcellaran, 20% *w*/*w* black currant juice and 80% *w*/*w* whey; F50_100 contained 0.50% *w*/*w* furcellaran, 100% *w*/*w* black currant juice and 0% *w*/*w* whey. *** Mean values within a column (the difference between blackcurrant juice level, comparing the same fermentation time) followed by different superscript letters statistically differ (*p* < 0.05). Mean values within a column (the difference between fermentation time, comparing the same blackcurrant juice level and furcellaran concentration) followed by different uppercase letters differ (*p* < 0.05).

**Table 5:** Results of the sensory analysis of the model samples (appearance, aroma, taste, off-flavour, and overall rating) *, **.

Sample	Appearance	Taste	Aroma	Off-Flavour	Overall Rating
F25_0	4 ^a,A^	4 ^a,A^	4 ^a,A^	3 ^a,A^	4 ^a,A^
F25_10	4 ^a,A^	4 ^a,A^	3 ^b,A^	3 ^a,A^	4 ^a,A^
F25_20	3 ^b,A^	3 ^b,A^	3 ^b,A^	2 ^b,A^	3 ^b,A^
F25_100	2 ^c,A^	5 ^c,A^	2 ^c,A^	1 ^c,A^	3 ^b,A^
F50_0	4 ^a,A^	4 ^a,A^	4 ^a,A^	3 ^a,A^	4 ^a,A^
F50_10	4 ^a,A^	4 ^a,A^	3 ^b,A^	3 ^a,A^	4 ^a,A^
F50_20	2 ^c,B^	2 ^d,B^	2 ^c,B^	2 ^b,A^	2 ^c,B^
F50_100	2 ^c,A^	4 ^a,C^	2 ^c,A^	2 ^b,B^	3 ^b,A^

* Values are presented as the median; F25_0 contains 0.25% *w*/*w* furcellaran and 0% *w*/*w* blackcurrant juice; F25_0 contained 0.25% *w*/*w* furcellaran, 0% *w*/*w* black currant juice and 100% *w*/*w* whey; F25_10 contained 0.25% *w*/*w* furcellaran, 10% *w*/*w* black currant juice and 90% *w*/*w* whey; F25_20 contained 0.25% *w*/*w* furcellaran, 20% *w*/*w* black currant juice and 80% *w*/*w* whey; F25_100 contained 0.25% *w*/*w* furcellaran, 100% *w*/*w* black currant juice and 0% *w*/*w* whey. F50_0 contained 0.50% *w*/*w* furcellaran, 0% *w*/*w* black currant juice and 100% *w*/*w* whey; F50_10 contained 0.50% *w/w* furcellaran, 10% *w*/*w* black currant juice and 90% *w*/*w* whey; F50_20 contained 0.50% *w*/*w* furcellaran, 20% *w*/*w* black currant juice and 80% *w*/*w* whey; F50_100 contained 0.50% *w*/*w* furcellaran, 100% *w*/*w* black currant juice and 0% *w*/*w* whey. ** Median values within a column (the difference between blackcurrant juice level, comparing the same fermentation time) followed by different superscript letters statistically differ (*p* < 0.05). Mean values within a column (the difference between furcellaran concentration, comparing the same blackcurrant juice level) followed by different uppercase letters differ (*p* < 0.05).

## Data Availability

The original contributions presented in the study are included in the article, further inquiries can be directed to the corresponding author.
